# Investigation of Perception of Quality of Life and Psychological Burden of Patients Undergoing Hemodialysis—Quality of Life of Hemodialysis Patients

**DOI:** 10.3390/nursrep13030112

**Published:** 2023-09-19

**Authors:** Nikos Rikos, Anna Kassotaki, Chara Frantzeskaki, Maria Fragiadaki, Andreas Mpalaskas, Georgios Vasilopoulos, Manolis Linardakis

**Affiliations:** 1 Department of Nursing, School of Health Science, Hellenic Mediterranean University, 71410 Heraklion, Greece; 2 Venizelio General Hospital of Heraklion, 71410 Heraklion, Greece; annakasswtaki42@yahoo.gr (A.K.); charaphrantzeskake@gmail.com (C.F.); maria.frangiadaki@gmail.com (M.F.); andreasmpalaskas@gmail.com (A.M.); 3 Department of Nursing, School of Health Sciences, University of West Attica, 12243 Athens, Greece; gvasilop@uniwa.gr; 4 Clinic of Social and Family Medicine, Department of Social Medicine, Faculty of Medicine, University of Crete, 70013 Heraklion, Greece; linman@med.uoc.gr

**Keywords:** hemodialysis, chronic kidney disease (CKD), quality of life (QOL), psychological states, depression, anxiety

## Abstract

Chronic kidney disease (CKD) has a significant impact on the life of patients undergoing chronic periodic hemodialysis. It negatively affects their social, economic and family status, and particularly their psychological well-being. The aim of this study was to investigate the perception of the quality of life (QoL) and psychological burden of patients undergoing hemodialysis. A cross-sectional study was conducted with 63 patients. Τhe majority were men (63.5%), and the mean age of the patients was 66.7 years (±12.9) with 61.9% aged 65–89 years. Data collection was performed in 2021 using the Hospital Anxiety and Depression Scale (HADS) and the Kidney Disease and Quality of Life-Short Form (KDQOL-SF™) research tools, and their relationships were assessed using parametric and non-parametric methods. Moderate to mild levels of Anxiety and Depression were found. Physical and Mental Composite Scores were mild to moderate, with the Mental Composite Score being significantly higher (*p* < 0.05). Anxiety and Depression were significantly correlated with lower QoL (*p* < 0.05), while a higher educational level was correlated with lower Depression Symptom Levels and higher QoL for Disease Symptoms, Disease Effects, Physical Functioning, Vitality and Overall Health (*p* < 0.05). A higher number of years of hemodialysis was correlated with lower levels of Anxiety and higher levels of Quality of Sleep (*p* < 0.05). Ease of Access to the Hemodialysis Unit was correlated with lower levels of Social Support (*p* < 0.05). The highest Mental Composite Scores were also correlated with a higher level of education, with patients living in urban areas, and with a higher monthly income (*p* < 0.05). Patients with moderate or severe levels of Anxiety and Depression had a lower QoL in the Physical and Mental Composite Scores, indicating their dependence on the appropriate medical, nursing and social environment in order to attain higher levels of well-being, leading to the improvement of patients’ health. This study was not registered.

## 1. Introduction

Quality of life (QoL) is an ambiguous and multifaceted term, which continues to be the object of research and dispute among many theoreticians due to its multivalency. Physical, mental and social well-being are considered the basic conditions for the fulfilment of human existence and a high QoL. The term “quality” defines the degree to which something can be perfect, while the term “life” defines the functionality and developmental ability of organisms. More generally, QoL can determine how a person functions in society, how they experience situations, how they perceive life values, and how their health itself can affect their life [1].

Because chronic kidney disease (CKD) is a progressive disease leading to irreversible loss of kidney function, it is a major public health problem worldwide [2]. It appears to be assuming epidemic proportions in developing countries, with increasing numbers of patients being treated using hemodialysis [2,3]. Among the main problems faced by patients suffering from CKD who are undergoing or about to undergo hemodialysis are their mental burden, stress, depression and, of course, the effect of all the related aggravating factors on their QoL, or even their difference from other patients with other diseases [4,5].

Chronic kidney disease (CKD) causes significant changes to the lives of patients undergoing chronic periodic hemodialysis, restricting or ending their professional career and changing their social and family roles due to the imposition of the role of patient. It negatively affects their social, economic and family status and particularly their psychological well-being. The changed body image brought about by the disease has a negative effect on a psychological, metabolic and functional level [6,7].

Despite the fact that in the initial stages of the disease, the effects on the general QoL of the patients are limited to discomfort, from the fourth stage of the disease onwards, the patients perceive an increasing amount of symptoms that can profoundly affect their daily life [8]. Fatigue, muscle weakness, cramps, itching, nausea and loss of appetite are reported as common symptoms. The already low QoL is further degraded in patients undergoing hemodialysis therapy. Conditions such as malnutrition, anemia, cognitive dysfunction, sleep disturbances, depression, reduced social interaction, physical and sexual dysfunction, and comorbidities such as diabetes and cardiovascular disease affect dialysis patients [8,9].

Limited self-care, physical and bodily deprivation, intense bodily pain, fatigue, limited social and family life, and low self-esteem in general are the main indications of diminished physical QoL. Frequent psychological distress, feelings of social isolation due to emotional problems, and low self-rating of mental health are the basic characteristics of poor mental QoL. The interrelationship between poor physical and poor mental QoL leads to depression in patients [10].

Several factors affecting aspects of health-associated QoL in CKD patients are noted in the international literature. The greatest differences have been observed in the aspects of physical functioning, vitality energy, mental health and general health [11,12]. These factors are particularly important due to the chronicity of the disease and the increased burden on patients caused by hemodialysis.

The aim of the present study was to investigate the perception of QoL and the psychological burden of patients undergoing hemodialysis, and to identify the factors affecting their level of health.

## 2. Materials and Methods

### 2.1. Study Design, Sample and Participants

The present study was a cross-sectional study using a questionnaire. The chosen sampling method was purposive sampling. The final study sample consisted of 63 patients. Their main characteristics were: (a) they were undergoing hemodialysis and were in end-stage renal failure, (b) they had the mental capacity to understand the aims and needs of the study, for example understanding and completing the questionnaire, and (c) they agreed to participate and complete the questionnaire after reading and signing the printed consent form. The study adhered to the Strengthening the Reporting of Observational studies in Epidemiology (STROBE) guidelines on cross-sectional studies.

### 2.2. Variables and Adequacy

A specialized instrument was used that measures the perceived QoL of patients undergoing hemodialysis, designed exclusively for patients undergoing periodic hemodialysis. It is directly available from the World Health Organization and is free for use by researchers for the better understanding of the psychosocial and physical conditions experienced by patients and for the continuous improvement of the health services provided [9].

The Kidney Disease and Quality of Life-Short Form (KDQOL-SF™-2009) questionnaire contains 19 components/subscales where the codified answers are rescaled to a scale of 1–100 [12,13,14,15,16]. The score is the weighted average of the questions comprising each component. Their reliability was assessed using Cronbach’s α with the exception of components consisting of one question (see Table 1). A higher score (closer to 100) indicates fewer symptoms or problems, better functioning or generally good QoL [7]. The second data collection tool was the Greek version of the Hospital Anxiety and Depression Scale (HADS). It consists of 14 items, each with 4 possible answers. It is designed to assess anxiety (HADS-A) and depression (HADS-D) (7 items for each, with a score ranging from 0 to 21). Permission to use the questionnaire was requested and granted [15].

### 2.3. Data Collection

Data collection was carried out at the Chronic Hemodialysis Unit “St. Panteleimon” Ltd. on the island of Crete over the course of two months, May and June 2021. The researchers met the participants inside the Chronic Hemodialysis Unit. For the purposes of the study, participating patients had to answer 24 questions on their medical history and QoL, and a set of questions including demographic data such as age and gender. Participants had the right to refuse to answer any question that might embarrass them or which they considered irrelevant to them. At no point was personal information, such as the patient’s name, requested, while the answers were completely confidential.

### 2.4. Ethical Considerations

Ethical approval was obtained from the Research and Bioethics Committee (IRB; Hellenic Mediterranean University, Crete, Greece 493/53/08-03-2021) and the Chronic Hemodialysis Unit “St. Panteleimon” Ltd. (Δ1_43/22-04-2021, Heraklion, Greece). The study participants were informed about the study objectives, expected outcomes, and associated benefits and risks. Written consent was received from the participants before they answered the questionnaire. The authors also obtained permission to use the hospital facilities before data collection.

### 2.5. Statistical Analysis

The analysis of the data in this study was performed using SPSS (IBM Corp. Released 2019, IBM SPSS Statistics for Windows, 25.0, Armonk, NY, USA: IBM Corp.). The frequency distributions of descriptive and other characteristics of the 63 study participants undergoing hemodialysis were calculated, presenting measures of central tendency, position and dispersion (mean, standard deviation, etc.). The shape of the distributions of the HADS and KDQOL-SF™ scores was reviewed using Blom’s method (Q-Q plot) and their reliability factors were calculated using Cronbach’s α. A strong asymmetry was observed in most components with the exception of the Physical and Mental Health components of the KDQOL-SF™ scale, which were compared using Student’s t-test. All the QoL and Anxiety/Depression components were then correlated to the patients’ characteristics using the non-parametric Spearman method, while the Physical and Mental Health components were correlated to the patients’ characteristics using Pearson’s method. The non-parametric Mann–Whitney U test was applied to compare the highest and lowest HADS Anxiety and Depression scores due to the small sample. The significance level was set at 0.05.

## 3. Results

### 3.1. Patient Characteristics

Of the 63 patients undergoing hemodialysis who participated in the study (Table 2), the majority were men (63.5%), and the mean age of the patients was 66.7 years (±12.9) with 61.9% aged 65–89 years. In total, 60.3% were married, 79.4% had children, and 6.3% had completed university education, with a further 6.3% holding a postgraduate degree. In addition, 95.2% were Greek and 81.0% were urban residents. The majority (50.8%) reported a monthly income of 1000+ euros, while 15.6% reported a monthly income of under 500 euros. The mean length of time they had been undergoing hemodialysis was 4.7 years (±4.4), with 61.9 years (±14.6) being the mean age of starting hemodialysis. Regarding access to the Hemodialysis Unit, 63.5% defined it as easy, while the majority (52.4%) said that they came by car (relatives). The frequency distribution of the reasons for hemodialysis of the 63 patients in the study (results not shown in figure/table) showed multiple causes, the most frequent being diabetic nephropathy (27.0%), followed by chronic kidney disease of unknown etiology (CKDu) (25.4%) and hypertension (19.0%).

### 3.2. Anxiety and Depression Symptoms in Patients

Table 3 presents the HADS scores of the 63 patients participating in the study. The mean Anxiety score is moderate to mild (7.7 ± 5.6), as is the Depression score (7.1 ± 4.7) and the total mean Anxiety and Depression score (14.8 ± 9.7). Generally, however, for both conditions, 54.0% of the patients were found to have normal scores or symptom levels. Cronbach’s α for both was 0.849–0.923, or excellent. However, between the moderate/severe levels of Anxiety and Depression (results not shown in figure/table), there was a non-significantly higher frequency of patients with moderate/severe levels of Anxiety (31.7%, 95%CI 20.6–44.4) vs. Depression (20.6%, 95%CI 11.1–31.7).

### 3.3. Patients’ QoL

Table 1 presents the scores of the KDQOL-SF™ scale and subscales or components of the questionnaire determining the QoL of the patients in the study. As the KDQOL-SF™ scale comprises many components divided into CKD or QoL, their reliability, assessed using Cronbach’s α, ranges from 0.536 to 0.947 (poor to excellent). Their mean scores also range from high to moderate or low, with a moderate Overall Health Rating (56.5). Of the other QoL components, the highest score and consequently the best quality was found for Social Functioning (61.5) and the lowest for Physical Role (38.1). Of the kidney disease components, the highest scoring was Hemodialysis Staff Encouragement (93.3) and the lowest was Patient Satisfaction (13.0). Table 1 also provides the frequencies of patients with the highest and lowest QoL score (with 0 and 100 as floor/ceiling, respectively). A high percentage of patients scored 0 in the components except for Patient satisfaction (60.3%), Work (68.3%) and Physical and Emotional Role (46.0%). The mean Physical and Mental Composite Scores were both low to moderate, at 37.6 (±10.5) and 44.0 (±11.7) respectively, with 0 and 100 as the floor/ceiling values and a higher score indicating better QoL. The mean Mental Composite Score was found to be significantly higher than the Physical Composite Score (*p* = 0.002), while Cronbach’s α for each was 0.763 and 0.839, respectively (excellent).

### 3.4. Characteristics, QoL, Anxiety and Depression

Of the univariate correlations between the KDQOL-SF™ questionnaire and the HADS subscales (results not shown in figure/table), the Anxiety and Depression subscales and the total of the two are significantly correlated with lower QoL in almost every subscale of the KDQOL-SF™ questionnaire. The highest correlations are between high Anxiety and low Cognitive Function (rho = −0.699, *p* < 0.05) or most Disease Symptoms (rho = −0.690, *p* < 0.05), and between high Depression Symptom levels and a low Overall Health Rating (rho = −0.791, *p* < 0.05) or low Vitality (rho = −0.738, *p* < 0.05). Furthermore, the Total Anxiety and Depression scale is correlated with the lowest Cognitive Function (rho = −0.753, *p* < 0.05) and the lowest Overall Health Rating (rho = −0.741, *p* < 0.05). Similarly, correlating the subscales of the KDQOL-SF™ questionnaire and the HADS, older patients are associated with lower levels of Sexual Function (rho = −0.270, *p* < 0.05), while those with children have a higher General Health Rating (rho = −0.249, *p* < 0.05). Higher educational level is associated with lower levels of Depression Symptoms (rho = −0.314, *p* < 0.05) and better QoL for Disease Symptoms (rho = 0.384, *p* < 0.05), Disease Effects (rho = 0.435, *p* < 0.05), Physical Functioning (rho = 0.518, *p* < 0.05), Vitality (rho = 0.364, *p* < 0.05) and Overall Health Rating (rho = 0.380, *p* < 0.05). Urban residents had better QoL for Disease Symptoms, (rho = 0.256, *p* < 0.05), Physical Role (rho = 0.343, *p* < 0.05) and Emotional Role (rho = 0.411, *p* < 0.05). Higher income is also correlated with lower Depression Symptom Levels (rho = −0.266, *p* < 0.05) and better QoL for Disease Burden (rho = 0.266, *p* < 0.05), General Health (rho = 0.353, *p* < 0.05) and Overall Health Rating (rho = 0.392, *p* < 0.05). More years of hemodialysis are correlated with lower levels of Depression (rho = −0.269, *p* < 0.05) and better Quality of Sleep (rho = 0.287, *p* < 0.05). On the contrary, a higher age of starting hemodialysis is associated with worse Quality of Sleep (rho = −0.255, *p* < 0.05), Physical Function (rho = −0.407, *p* < 0.05) and Emotional Role (rho = −0.276, *p* < 0.05). Finally, Ease of Access to the Hemodialysis Unit is correlated with lower levels of Social Support (rho = 0.265, *p* < 0.05).

The main components of the QoL SF-12 scale, estimated using the KDQOL-SF™ (Table 4), show that higher Physical Composite Scores are associated with younger patient ages (rho = −0.377, *p* < 0.05), higher educational level (rho = 0.475, *p* < 0.05), younger ages of starting hemodialysis (rho = −0.267, *p* < 0.05) and lower levels of Depression Symptoms (rho = −0.377, *p* < 0.05). The best Mental Composite Scores are also associated with higher educational levels (rho = 0.357, *p* < 0.05), with patients living in urban areas (rho = 0.348, *p* < 0.05) and with a higher monthly income (rho = 0.257, *p* < 0.05).

Finally, Table 5 compares the scores of the two main components of the QoL SF-12 scale for the 63 study participants to the mild and severe levels of Anxiety and Depression according to the HADS. It is generally observed that patients with moderate or severe Anxiety or Depression have a lower QoL in the Physical and Mental Composite Scores (*p* < 0.01). Patients with moderate or severe Anxiety have a lower mean Physical Composite (32.0 vs. 40.2, *p* = 0.007) or Mental Composite Score (34.3 vs. 48.5, *p* = 0.008) than patients with normal/mild Anxiety. Furthermore, patients with moderate or severe Depression have a lower mean Physical Composite (30.7 vs. 39.4, *p* < 0.001) or Mental Composite Score (32.6 vs. 46.9, *p* < 0.001) than patients with normal/mild Depression.

## 4. Discussion

The aim of the present study was to investigate the perception of the QoL and psychological burden of patients undergoing hemodialysis, and to identify the factors affecting their level of health.

In brief, the following findings emerged. Moderate to mild Anxiety and Depression scores were noted. The QoL components scored low to high, with a moderate Overall Health Rating, while Social Functioning scored the highest and Physical Role scored the lowest. Of the CKD components, the highest scoring was Hemodialysis Staff Encouragement and the lowest was Patient Satisfaction. The Physical and Mental Composite Scores were low to moderate, with the Mental Composite Score being significantly higher (*p* < 0.05). The Anxiety and Depression scores are significantly correlated with lower QoL in almost all the subscales of the KDQOL-SF™ questionnaire (*p* < 0.05). A higher educational level is correlated with lower Depression Symptom Levels and higher QoL for Disease Symptoms, Disease Effects, Physical Functioning, Vitality and Overall Health (*p* < 0.05). Higher income is correlated with lower Depression Symptom Levels and better QoL for Disease Burden, General Health and Overall Health Rating (*p* < 0.05). A higher number of years of hemodialysis is correlated with lower levels of Anxiety and higher levels of Quality of Sleep (*p* < 0.05). A higher age of starting hemodialysis, on the contrary, is correlated with lower levels of Quality of Sleep, Physical Functioning and Emotional Role (*p* < 0.05). Ease of Access to the Hemodialysis Unit is correlated with lower levels of Social Support (*p* < 0.05). The highest Mental Composite Scores are also correlated with a higher level of education, with patients living in urban areas, and with a higher monthly income (*p* < 0.05).

One of the main findings of the present study was the fact that patients with moderate or severe levels of Anxiety and Depression have lower QoL in Physical and Mental Health Composite Scores (*p* < 0.01). Generally, however, large-scale population studies have shown that Physical Health is at much lower levels than Mental Health [17], with the disease being considered almost entirely responsible for this.

These results, however, are not a new development according to the current state of knowledge of the literature, as many studies have sought to link the mental stress caused by the disease and/or the treatment (hemodialysis) to patients’ quality of daily life. In Greece, in the relatively distant past, Kontodimopoulos and Niakas (2005) [16], adapting the KDQOL-SF to Greek conditions, studied the QoL of 665 patients undergoing hemodialysis, representing about 10% of CKD patients in the country. They found a mean of just 36.3 ± 21.7 for the general health component, while Malindretos and colleagues (2010) [13] also found a mean of <35.0, levels that were naturally lower than those of the present study (41.1 ± 21.1). Using a different assessment tool, Garofyllou and colleagues (2017) [17] found moderate mean levels of Physical Health in Greek hemodialysis patients. In any case, these findings may indicate low to moderate QoL, while a change over time may be apparent due to changes in, for example, living conditions, study samples, hemodialysis or comorbidity, and stage of disease.

For example, on the level of individual studies, Takahashi and colleagues (2020) studied 55 patients who had been undergoing hemodialysis for over a year [18]. Using similar assessment tools to the present study, the HADS and the KDQOL-SF, they used combined frequency results to group the patients into two groups: (a) those who took on “unstable psychological conditions” if the HADS indicated that “depression is suspected” or “suspected depression, anxiety stated” (“Anxiety/Depression group”), and (b) those who were stable regarding the relevant outcomes (absence of depression and anxiety symptoms). A total of 32.7% patients were found to present symptoms of Anxiety/Depression (“Anxiety/Depression group”), while they had significantly lower QoL in components such as “effects of kidney disease”, “social support” and “general health perceptions”. Similarly, in the present study, Anxiety and Depression were treated separately using the HADS, while the analysis focused on the two main QoL components, the Physical and Mental Composite Scores, which were shown to be significantly lower in patients with moderate or severe Anxiety and Depression.

The same conclusion was reached by another study on 100 patients in Brazil [2], where a negative correlation was found between Anxiety and Depression and QoL, while the mean KDQOL-SF scores were significantly lower for patients with symptoms of Anxiety and Depression. Specifically assessing the two domains of Physical and Mental Health, Ademola and colleagues (2020) in Nigeria [3], comparing 150 CKD patients with 150 healthy individuals, found that the patients had lower scores in both (*p* < 0.05), while they also observed a progressive decline in the scores of all KDQOL components with advancing CKD stages (*p* < 0.05).

A study in France similarly correlated QoL to the stage of the disease, while patients undergoing hemodialysis seemed to have generally lower QoL than the general population [19]. Li and colleagues (2016) went a step further in their Los Angeles study [20], assessing 72 patients who had been on dialysis for over six months, compared to 39 normal controls of similar age and gender distribution. The researchers found that most of the KDQOL components were lower in the group of 72 patients, while daily exercise was positively correlated with certain KDQOL scores. The consideration of lifestyle factors, their absence from or presence in patients’ daily lives, obviously provides valuable information on patients’ QoL, but unfortunately, these could not be included in the present study due to an excess of informational material.

In a systematic overview of the role of depression, anxiety and adherence to treatment on the QoL of hemodialysis patients, 100.0% of studies found a negative correlation between these factors and QoL [21]. Adherence to treatment also appeared to be associated with psychological factors, while the overview generally considered that screening for the early indicators of factors affecting QoL, such as stress, depression or difficulties in complying with treatment, can help improve patients’ health.

In their study on 183 patients in Brazil, Pretto and colleagues (2020) also attempted to include variables that could be related to QoL [22]. These included sociodemographic and clinical characteristics, depression symptoms and medication adherence characteristics. They found moderate QoL scores, with low scores being associated with repetitive infections and edema as complications of the disease, pain during hemodialysis and weakness afterwards, anemia and low medication adherence.

It therefore seems that patients’ QoL is determined by the above factors, while the appropriate actions by their supporting medical, nursing and social environment can achieve the desired well-being and mitigation of complications.

Similarly, a 2023 study by Alshelleh and colleagues in Indonesia found that the majority of participants (92.4%) had depression and 83.3% had generalized anxiety disorder, factors that severely impacted their QoL [23].

The present study was carried out during the second wave of the COVID-19 pandemic. Comparing the results of this study with previous studies in Greece and abroad, no significant difference in patients’ QoL was observed, either in their physical or their mental condition, which would indicate that the pandemic significantly impacted their QoL. A search of the literature for corresponding studies during this time period found similar results to those of the present study. Ashgar et al. (2022) observed low QoL (*p* > 0.001) in all subscales (*p* = 0.003) [24].

Yun Szu and colleagues (2023) also noted similar results, with poorer QoL in hemodialysis patients with mild, moderate, or severe anxiety [25]. In the study by Segura-Ortí and colleagues in 2022, the linear regression model showed that the pandemic had a significant impact on health-related QoL (HRQoL). Physical function (−15.7) and social functioning subscales (−28.0) worsened (*p* = 0.001), and the physical component scale also showed a significant decrease (−3.6; *p* = 0.05). Nevertheless, there are no significant divergences from the results of the present study [26].

Finally, the study by Nadort and colleagues (2022) found that patients impacted by COVID-19 had higher stress levels (mean difference (MD) 4.7 (95%CI 1.5; 8.0), *p* = 0.005) and BDI-II scores (MD 4.9 (95%CI 0.7; 9.0), *p* = 0.021) and lower SF-12 mental component summary scores (MD 5.3 (95%CI 9.0, −1.6), *p* = 0.006) than patients who did not experience COVID-19 stress. These differences were already present before the pandemic, and the results of this study are also not significantly different from ours [27].

## 5. Limitations

The present study, like any research effort, contains weaknesses that may technically prevent the correct assessment of its findings. However, in the context of a clinical environment under conditions of strict lockdown protocols due to the COVID-19 pandemic, the researchers did all they could to utilize all the opportunities provided. The main weakness was the small sample of selected patients, which was determined by the influx into the Hemodialysis Unit of the hospital, while no other unit could be used due to the pandemic and the lockdown measures. Another weakness was the lack of a control group allowing comparison of QoL, Anxiety or Depression with the patient group, as often referenced in the literature [3,4,5,20,21]. The impact on the QoL of carers or other patients could also have been taken into account in order to verify the findings. Moreover, the overall picture of the impact factors should include lifestyle risk factors or basic habits such as lack of exercise, smoking, alcohol consumption or poor diet [21,22].

## 6. Conclusions

The aim of the present study was to investigate the perception of the QoL and the psychological burden of patients undergoing hemodialysis. The participants were found to have moderate to mild levels of Anxiety and Depression and low to high QoL component scores. Their Physical and Mental Composite Scores were low to moderate, with the Mental Composite Score being significantly higher in relation to the level of education, living in urban areas and higher monthly income (*p* < 0.05). Moreover, patients with moderate or severe Anxiety and Depression had lower QoL and lower Physical and Mental Composite Scores (*p* < 0.01), indicating their dependence on the appropriate supporting medical, nursing and social environment in order to attain higher levels of well-being, leading to the improvement of patients’ health.

## Figures and Tables

**Table 1:** KDQOL-SF™ questionnaire and QoL SF-12 scale/subscale or component scores determining the QoL of the 63 study participants.

QoL Components	Mean	SD	% Floor	% Ceiling	Cronbach’s α
Kidney disease-targeted scales ^a^					
Disease symptoms	70.7	20.7	-	4.8	0.793
Disease effects	55.4	23.3	-	1.6	0.847
Disease burden	40.8	25.7	6.3	4.8	0.683
Work status	17.5	27.2	68.3	3.2	0.539
Cognitive function	73.5	26.7	-	27.0	0.897
Quality of social interaction	67.4	21.3	-	7.9	0.558
Sexual function	48.2	35.1	22.2	17.5	0.920
Quality of Sleep	51.6	21.5	1.6	-	0.774
Social support	78.0	30.9	6.3	54.0	0.861
Hemodialysis staff encouragement	93.3	13.6	-	74.6	0.851
Patient satisfaction	13.0	21.9	60.3	3.2	-
36-Item Health Survey scales					
Physical functioning	45.9	33.9	14.3	3.2	0.947
Physical role	38.1	43.7	46.0	28.6	0.919
Bodily pain	60.4	32.6	4.8	22.2	0.918
General health	41.1	21.1	-	-	0.692
Emotional well-being	57.3	17.7	-	1.6	0.585
Emotional role	46.6	47.3	46.0	41.3	0.938
Social functioning	61.5	29.2	3.2	20.6	0.684
Vitality	48.5	17.1	-	-	0.536
Overall health rating	56.5	22.0	-	9.5	-
QoL SF-12 ^b^					
Physical Composite Score	37.6	10.5	-	-	0.839
Mental Composite Score	44.0	11.7	-	-	0.763

^a^ Higher scale → lack of symptoms or better QoL. ^b^ Higher score (→100) indicates higher QoL. Student’s *t*-test between the two subscales, *p* = 0.002.

**Table 2:** Descriptive characteristics of the 63 study participants undergoing hemodialysis.

		*n*	%
Gender	Men/Women	40/23	63.5/36.5
Age, years	Mean age ± SD	66.7 ± 12.9	
	22–64	24	38.1
	65–86	39	61.9
Marital status	Unmarried	10	15.9
	Divorced	5	7.9
	Widowed	10	15.9
	Married, with a partner	38	60.3
Children	Yes	50	79.4
Educational level	Primary school	23	36.5
	Lower secondary school	11	17.5
	Higher secondary school	21	33.4
	University, technical college	4	6.3
	MA, MSc, PhD	4	6.3
Nationality	Greece	60	95,2
Place of residence	Rural	12	19.0
	Urban	51	81.0
Monthly income, euros	<500	10	15.9
	500–1000	21	33.3
	1001–1500	23	36.5
	>1500	9	14.3
Years undergoing hemodialysis	Mean ± SD	4.7 ± 4.4	

SD, standard deviation.

**Table 3:** HADS scores of the 63 study participants.

	Mean	SD	Median	Min.	Max.	Cronbach’s α
HADS Anxiety (higher score → higher symptom levels)	7.7	5.6	6.0	0	19	0.892
normal (<8)	*n* = 34 or 54.0%					
mild (8–10)	*n* = 9 or 14.3%					
moderate (11–14)	*n* = 12 or 19.0%					
severe (15–21)	*n* = 8 or 12.7%					
HADS Depression (higher score → higher symptom levels)	7.1	4.7	7.0	0	21	0.849
normal (<8)	*n* = 34 or 54.0%					
mild (8–10)	*n* = 16 or 25.4%					
moderate (11–14)	*n* = 9 or 14.3%					
severe (15–21)	*n* = 4 or 6.3%					
HADS Total (higher score → higher symptom levels)	14.8	9.7	14.0	0	40	0.923

**Table 4:** Correlation of characteristics and HADS scores with QoL SF-12 scores of the 63 study participants (assessed using KDQOL-SF™).

	SF-12	
	Physical Composite Score	Mental Composite Score
	r-Pearson	
Gender (1: men, 2: women)	−0.082	−0.192
Age (years)	−0.377 *	−0.123
Marital status (1: unmarried, divorced, widowed, 2: married, with a partner)	−0.017	−0.083
Children (1: yes, 2: no)	−0.104	−0.074
Education (1: primary school, 2: lower secondary school, 3: higher secondary school, 4: university, technical college, 5: MA, MSc, PhD)	0.475 *	0.357 *
Place of residence (1: rural, 2: urban)	0.176	0.348 *
Monthly income (1: < EUR 500, 2: 500–1000, 3: 1000–1500, 4: >1500)	0.211	0.257 *
Years of hemodialysis	−0.233	0.044
Age of starting hemodialysis (years)	−0.267 *	−0.132
Access to the unit (1: easy, 2: neither easy nor difficult, 3: difficult)	−0.091	−0.098
HADS anxiety	−0.082	−0.192
HADS depression	−0.377 *	−0.123
HADS total	−0.017	−0.083

* *p* < 0.05.

**Table 5:** Comparison of scores of the two main components of the QoL (SF-12) Scale for the 63 study participants for mild and severe levels of Anxiety and Depression (HADS).

		QoL							
		Physical Composite Score				Mental Composite Score			
HADS Levels		*n*	Mean	SD	*p*-Value	*n*	Mean	SD	*p*-Value
Anxiety	normal or mild (score up to 10)	43	40.2	10.2	0.007	43	48.5	10.4	0.008
	moderate or severe (>10)	20	32.0	9.1		20	34.3	73.6	
Depression	normal or mild (up to 10)	50	39.4	10.0	<0.001	50	46.9	10.8	<0.001
	moderate or severe (>10)	13	30.7	10.1		13	32.6	7.1	

Anxiety and Depression scores were classified in 4 categories: normal (score < 8), mild (8–10), moderate (11–14) and severe (15–21). SD, standard deviation. Mann–Whitney tests.

## Data Availability

The data used in this study are not open to other researchers at this time.

## Public Involvement Statement

No public involvement in any aspect of this research.

## Guidelines and Standards Statement

The study adhered to the Strengthening the Reporting of Observational studies in Epidemiology (STROBE) guidelines on cross-sectional studies.
